# Legal regulation of surrogacy parentage determination in China

**DOI:** 10.3389/fpsyg.2024.1363685

**Published:** 2024-09-06

**Authors:** Wenting You, Jun Feng

**Affiliations:** ^1^School of Law, Shanxi University, Taiyuan, China; ^2^School of Biological Sciences and Technology, Taiyuan Normal University, Jinzhong, China

**Keywords:** Chinese civil code, full surrogacy, partial surrogacy, international surrogacy, Chinese regulations

## Abstract

Unlike natural conception and other assisted reproductive technologies, surrogacy involves three crucial factors: family legislation, family ethics, and reproductive technology. This makes the determination of parentage in surrogacy more complex. In China, surrogacy is completely prohibited by law. However, this prohibition has not diminished the interest in discussions around the family ethics, order, and relationships affected by surrogacy. In practice, disputes over parentage and child custody arising from surrogacy urgently need resolution through judicial practice. The current legal framework in China lacks clear regulations to address the complexities of surrogacy, leading to numerous unresolved disputes. To address this issue, it is advisable for China to enact clear legislative measures to govern parent–child relationships in surrogacy cases. This paper presents legislative recommendations for regulating surrogacy in China, with the hope that the judicial interpretations of the Supreme People’s Court of China can provide clear legal regulations on surrogacy during revisions.

## Introduction

At the core of the family lies the parent–child relationship, serving as the bedrock of this societal unit. In the face of rapid technological advancements, family structures have evolved, drawing increased attention from various sectors of society to the diverse parent–child relationships formed through surrogacy.

Surrogacy explores a significant market demand in China. According to a report by the World Health Organization (WHO) on April 4, 2024,^1^ there are 48 million couples and 186 million individuals worldwide suffering from infertility, affecting approximately one in every six people. “A Lancet Commission on 70 years of Women’s Reproductive, Maternal, Newborn, Child, and adolescent Health in China” indicated that infertility affects 10% of married couples in China (Qiao et al., 2021), doubling from 4.8% reported in 1984. The incidence of infertility is on the rise, with approximately one in every seven couples facing reproductive challenges. Clinical statistics show that about 20% of infertile couples are unable to conceive without the assistance of reproductive technologies.

Zhou Qiang, the former Chief Justice of the Supreme People’s Court of China, specifically highlighted the nation’s first dispute over guardianship rights involving surrogate children during the third plenary session of the twelfth National People’s Congress held in March 2017 (Author, 2020). This case went through two trials at the People’s Court of Minhang District, and the Intermediate People’s Court of Shanghai. Ultimately, based on the principle of the Best Interests of the Children, the mother was determined as the guardian of the surrogate twins. This decision attracted widespread attention nationwide.

Surrogacy in China continues to proliferate despite repeated bans. As of April 9, 2024, a search on the China Judgments Online^2^ using the keyword “surrogacy” yielded 486 court judgments. These cases primarily revolve around contract disputes, accounting for 49.4%, and disputes related to parent–child relationship determinations, accounting for 11.1%. The term “infertility” appeared 189 times in these cases, indicating a significant number of people seeking surrogacy due to infertility, which often leads to disputes. The considerable prevalence of surrogacy in China underscores the urgent need for legal regulation.

The regulation and clarification of parent–child relationships resulting from surrogacy are essential. Much like *in vitro* fertilization, surrogacy represents a key component of modern assisted reproductive technology.

Article 1009 Medical and scientific research activities concerning human genes and human embryos, among others, shall be carried out according to the laws and administrative regulations, and relevant provisions issued by the state, without endangering human health, violating moral principles, or damaging public interests.

In accordance with Article 1,009 of the Chinese Civil Code, China currently lacks a comprehensive legal framework for surrogacy. Dependence solely on longstanding administrative regulations may not offer a sustainable resolution to this societal matter. As surrogacy is experiencing standardized and widespread growth, it becomes imperative for Chinese law to specifically address the intricacies associated with surrogacy.

Currently, countries worldwide generally adopt three main legislative stances toward surrogacy. The first is complete support, exemplified by Israel, furthermore, most Eastern European countries allow commercial surrogacy, which has led countries like Ukraine to be dubbed as global Surrogacy Factories by many media outlets. The second is complete opposition, such as some European countries, represented by France, and Middle Eastern countries, represented by the United Arab Emirates. The third is conditional support, as observed in the United States. While many U.S. states have laws either prohibiting or imposing restrictions on surrogacy, partial surrogacy has become increasingly prevalent and socially accepted since the late 20th century, owing to advancements in assisted reproductive technologies. In some states such as Florida and Nevada, surrogacy agreements are recognized as legal, but only if they involve reasonable compensation for surrogacy services. States like Washington and Arizona deem surrogacy agreements invalid and unenforceable, while California maintains a fully open stance toward commercial surrogacy. As a typical example allowing only altruistic surrogacy, the Human Fertilization and Embryology Act of 1990, enacted in the United Kingdom, stipulates that surrogacy must be licensed by the statutory regulatory authority for surrogacy, the Human Fertilization and Embryology Authority, placing surrogacy under legal jurisdiction through proactive prevention measures.

China takes a stance of complete opposition to surrogacy. Article 3 of the Regulations on the Management of Human Assisted Reproductive Technology, issued by the former Ministry of Health of China on February 20, 2001 (now the National Health Commission of the People’s Republic of China), prohibits medical institutions and healthcare professionals from practicing any form of surrogacy technology. On April 3, 2015, the Publicity Department of the Communist Party of China Central Committee, in conjunction with the General Office of the National Health and Family Planning Commission and nine other State Council departments, issued a Notice on Issuing the Work Plan for Carrying Out Special Actions to Combat Surrogacy. The nationwide special action aimed to address prominent issues related to surrogacy.

From June to December 2023, 14 departments, including the National Health Commission and the Central Political and Legal Affairs Commission, jointly issued the Work Plan for Conducting Special Actions to Combat the Illegal Application of Human Assisted Reproductive Technology. The plan aims to conduct rigorous nationwide campaigns to combat the illegal application of human assisted reproductive technology, regulate its application, and severely crack down on various illegal and criminal activities arising from it.

Public opinions on surrogacy vary widely, and the Chinese government lacks comprehensive legal regulation on this practice (Liang, 1993; Liu C. 2016; Zhang, 2018; Luo, 2009; Anderson, 2020; Satz, 1992; Lin and Huang, 2011; Casey et al., 2016; Ge, 2001; Li, 2005; Lozanski, 2015). Opposition to the legalization of surrogacy often centers on the argument that it Objectifies Women and reduces them to mere Baby-making Machines (You, 2022; Spector, 2016; Field, 2014; Lin and Huang, 2011; Liu, 2004; Li, 2008; Liu, 2016a). However, a minority of people believe that legalizing surrogacy can serve as a regulatory measure, ensuring that the surrogacy market operates in an orderly manner (Zheng, 2019; Cao, 2012; Zhang, 2007; Mei and Xu, 2015).

Globally, there is opposition to surrogacy, primarily based on moral considerations (Scott, 2009; Ford, 2008; Lozanski and Shankar, 2019; Epstein, 1995; Berkhout, 2008; Foret and Bolzonar, 2021; Weiss, 1992). However, this article does not focus on whether surrogacy aligns with moral standards. Instead, it primarily explores how legal conflicts surrounding surrogacy can be effectively resolved. Countries can address surrogacy by enacting clear domestic legislation or by recognizing foreign laws without the need for additional domestic regulation.

Divergent legal provisions across countries exacerbate conflicts between domestic and foreign laws, placing surrogate mothers, intending couples, and surrogate-born children in contentious positions. These entities often find themselves navigating a gray area of international legal standards. The establishment of uniform international rules through international conventions or bilateral agreements can not only address legislative gaps but also rationalize the contentious interests of surrogate parties.

It’s apparent that Chinese law has largely avoided addressing the issue of surrogacy. Aside from the administrative regulation known as the “Regulations on Assisted Reproductive Technologies,” which expressly prohibits medical institutions and personnel from participating in surrogacy, there are no specific prohibitions on other individuals engaging in surrogacy. However, regardless of the legal status of surrogacy, the parent–child relationship of surrogate children cannot be disregarded. It’s unacceptable to deny a child’s parental rights simply because surrogacy may be illegal. Therefore, the law should provide a clear and decisive response, avoiding ambiguity.

The legal contradictions have led to divergent views on the legitimacy of surrogacy itself. In reality, the path to legalizing surrogacy in China remains unclear, and there are valid arguments within the legal community. However, the contradictions in legal theory are also a significant factor. The narrow scope and limited applicability of China’s laws prohibiting surrogacy, which primarily target medical personnel, leave informal surrogacy arrangements unregulated, creating a legal Grey Area. Moreover, regarding the regulation of surrogacy contracts, the provisions on public order and good customs in the Chinese Civil Code (Article 8) lack specificity. This provision, as an integral part of the legal system, relies on legal techniques for implementation, resulting in less-than-ideal legal effectiveness.

Both domestic and international literature on surrogacy primarily focuses on the legality of surrogacy. Most scholars concentrate on arguing why and how surrogacy should be legalized (Lin and Huang, 2011; Ren, 2015; Wang and Luo, 2009; Gao, 2008; Casolo et al., 2019; Berk, 2015). They either do not elaborate on the issue of determining the parent–child relationship of surrogate children or merely use it as a supporting argument for the legalization of surrogacy, without conducting further in-depth research on this matter. In reality, whether surrogacy is legal or not does not affect the legitimate existence of surrogate children, nor does it affect their independent and complete personality rights. Regarding surrogacy, what the Chinese law needs to address is the issue of determining the parent–child relationship of surrogate children after their birth.

There is currently no comprehensive legal framework regulating surrogacy in China. The Civil Code lacks clear regulations concerning rights and obligations regarding assisted reproductive technologies like surrogacy, resulting in numerous unresolved disputes in surrogacy cases. Surrogacy is currently undergoing standardized and large-scale development, and relying solely on administrative regulations for long-term control is not a sustainable solution to address this social issue. Therefore, it is imperative for Chinese law to address the issue of surrogacy. Therefore, it is advisable for the Civil Code to explicitly outline the parent–child relationship of surrogate children.

In 2022, China’s National Health Commission, along with 17 other departments, jointly issued the “Guiding Opinions on Further Improving and Implementing Supportive Measures for Positive Birth,” which proposes the enhancement of eugenics services in medical institutions through assisted reproductive technologies. As China gradually relaxes its birth policy and introduces supportive measures, issues related to infertility and difficulties in conception have gained increased attention. The Chinese government is progressively altering its stance on assisted reproductive technologies, potentially relaxing restrictions on surrogacy through legislative revisions. Therefore, this article presents legislative recommendations for the regulation of surrogacy and offers theoretical suggestions for the future amendment of China’s Civil Code.

The Chinese Civil Code does not provide regulations for surrogacy, leading to the proliferation of “underground surrogacy” and persistent issues despite prohibitions. The law must confront these problems directly. This paper aims to propose amendments to the judicial interpretations of the Civil Code, recommending that the Supreme People’s Court of China explicitly regulate surrogacy in its revisions. This study employs a normative analysis approach in legal interpretation, serving to address and supplement the existing legal gaps.

## The necessity of regulating the surrogacy parent–child relationship in China

China’s legal stance on surrogacy is ambiguous and evasive. As early as 2003, the Ministry of Health issued the “Regulations on Human Assisted Reproductive Technology,” which prohibited surrogacy. However, due to its low legal status, limited scope of application, and lack of supporting systems, this regulation has had little effect on controlling surrogacy. The inadequacy of legislation has led to the increasing proliferation of the surrogacy “black market.” Relevant regulations could have been elevated to a higher legal status through the Population and Family Planning Law, gaining more regulatory effectiveness. However, the Prohibition of Surrogacy clause was removed from the revised text of the Population and Family Planning Law passed by the National People’s Congress Standing Committee in December 2015.

In summary, the legislative hesitation reflects the lack of a broad consensus in society on whether surrogacy should be completely banned or moderately permitted. Currently, legislation regulating surrogacy is at a crossroads, facing many uncertainties. As the demand for surrogacy increases, the resulting legal complexities and heightened social tensions can no longer be ignored. Determining the future regulatory approach for surrogacy has become a significant responsibility for the legal field and scholars. This paper provides legislative suggestions for the judicial interpretation of the Civil Code and conducts an in-depth analysis of surrogacy regulation models, aiming to address and fill the gaps left by previous theoretical and practical research.

### The development of assisted reproductive technologies necessitates laws to keep pace with the times

Initially, surrogacy encountered significant resistance in Chinese society due to its clash with traditional family values, leading to a prolonged period of rejection and denial. This conceptual resistance hindered the integration of surrogacy into mainstream and organized social life. Surrogacy persists on the social periphery, facing rejection on one hand while flourishing unrestricted on the other. With the increasing prevalence of surrogacy-related issues, there is an urgent need for legal regulation. This regulation requires not only amendments to marital and family laws but also coordination with various legal domains, including contract law and inheritance law.

Different branches of law deal with different issues of surrogate paternity. For example, in the contract law, the parties involved, surrogacy participants are regarded as equal parties. Legal relationships, surrogacy contracts are seen as contractual agreements. In the general provision of the civil code, concerning actions, surrogacy is categorized as a civil legal act. Therefore, surrogacy falls under the purview of civil law and is governed by the Civil Code. While in the succession law, marriage and family law oversee the parent–child relationship in surrogacy, it dictates the distribution of assets following the death of one or both parties of the intending couple during or after pregnancy. As like Article 1,155 of the Chinese Civil Code mandates that a portion of the inheritance be set aside for the unborn child.

Article 1155 At the time of the partitioning of the estate, reservation shall be made for the share of an unborn child. The share reserved shall be dealt with in accordance with provisions on statutory succession if the baby is stillborn.

This provision equally extends to the fetus in surrogacy, although its legal acknowledgment of the parent–child relationship with the intending couple may not be recognized. This situation could potentially lead to the inability to assert inheritance rights for the child born via surrogacy (Xue, 2020). The Contract Law governs aspects such as the non-commercial nature of contracts, the involved parties, contract contents, and the enforceability of contracts. Therefore, the regulation of surrogacy should emerge from a systematic and comprehensive approach within civil law. Indeed, any legal outcome should arise from a thorough evaluation of multiple laws, which is also the explicit aim of the revision of the Chinese Civil Code.

With advancements in life science and technology, as well as evolving societal norms, novel methods of establishing parent–child relationships have emerged. These new dynamics in parent–child relationships present greater demands on both legal and moral considerations (Li, 2008). Due to the unpredictable consequences of open surrogacy, the law can only strictly regulate and provide a cautious assessment. Currently, China’s stance on surrogacy has softened, as the ‘Law on Population and Family Planning’ removed the prohibition of surrogacy during its revision in 2016. The Marriage and Family Book of Civil Code Draft (Second Revision) initially proposed recognizing the parent–child relationship between children born through assisted reproductive technologies and the couples opting for such methods. Although this provision was subsequently removed upon formal promulgation, the regulatory concerns surrounding surrogacy relationships have become a focal point for the Chinese legislature.

Within the Chinese Civil Code, strive to harmonize legislative regulations within the domain of civil law to comprehensively and systematically address surrogacy through contract law, family law, succession law, and other pertinent legal sectors. Adopt a three-dimensional, rolling legislative approach to establish a systematic and coherent framework. If the Chinese Civil Code governs surrogacy, it not only demonstrates the necessity for legal evolution but also enhances alignment with international attitudes to surrogacy more effectively.

### The acknowledgment of international surrogacy parent–child relationships necessitates a robust domestic legislative framework

The surrogacy issue extends beyond national boundaries, posing challenges not only domestically but also internationally. Both the surrogate mother’s home country and the intending parents’ nation face potential risks. Individuals involved in international surrogacy agreements may encounter disputes arising from cultural, political, religious, economic, and regional differences, which could escalate into international conflicts involving nations, regions, or ethnic groups. Additionally, women in economically disadvantaged countries who serve as surrogates may risk commodification, while intending couples from more affluent nations may inadvertently expose them to personal and economic exploitation, including instances of racial and gender discrimination.

The challenges posed by surrogacy are heightened on the international scale. Varying legal approaches to surrogacy among different nations exacerbate international legal disputes. Intending couples may face obstacles when endeavoring to repatriate children born through surrogacy in foreign jurisdictions to their home country, where the child’s status may not be recognized, leaving them stateless. In their efforts to formalize the parent–child relationship with surrogate-born children, many intending couples resort to extreme measures, occasionally involving illicit methods (You, 2016). Despite the significant expenses associated with surrogacy, participants persist in seeking legal regulation for the practice, even if it entails certain legal limitations.

Governments worldwide often take a hands-off approach to surrogacy, highlighting the urgent need for a globally unified framework. In June 2010, The Hague Conference deliberated on the legal regulation of surrogacy but ultimately decided against establishing international norms for surrogacy legislation. As a result, surrogacy has neither been expressly prohibited by the international community nor officially recognized as a legitimate practice (Hou, 2016). The most relevant existing international legal framework concerning surrogacy is found in the provisions of the 1993 Hague Convention on Protection of Children and Co-operation in Respect of Intercountry Adoption. This convention serves as a guiding norm for international surrogacy. Globally, more than 80 countries have ratified, agreed to, or recognized this convention, including countries like the United States and India that adopt a permissive stance toward surrogacy. Fundamentally, the resolution of cross-border surrogacy issues relies on establishing a domestic legal foundation; otherwise, alignment with international law becomes challenging.

### The existing legal regulations in China are inadequate, calling for a systematic integration within the legal code system

#### The legislative contradictions in determining surrogacy parent–child relationships

The inadequacy of regulating surrogacy through the Regulations on Assisted Reproductive Technology in China stems from its classification as an administrative regulation, issued by a department of the Chinese State Council (The Regulation of the People’s Republic of China on the Administration of Human Genetic Resources, 2019). Administrative regulations, being of lower hierarchical rank than Laws, have a narrower scope and solely prohibit medical personnel from engaging in surrogacy. However, non-medical surrogacy remains unregulated by this administrative document. Administrative regulations lack the authority to govern surrogacy contracts, necessitating precision in legal regulation achieved through the legal system’s mechanisms. Moreover, relying on contract law to regulate surrogacy contracts may result in conflicts with other laws of equal rank, as detailed in Table 1.

**Table 1:** Contradictions in the legislative system of surrogacy.

Contradictions	Contents	
System	Article 3 and Article 22 of the ‘Regulations on Assisted Reproductive Technology’ prohibit medical institutions and medical personnel from conducting surrogacy.	Article 7 of the ‘Legislation Law’ stipulates the principle of legal reservation, requiring explicit legal authorization for restrictions on fundamental rights.
Principle	The Contracts Book of the Civil Code upholds the principle of freedom of contract.	Article 8 of the ‘General Provisions of the Civil Code,’ the principle of public order and good customs.
Provision	Article 3 and Article 22 of the ‘Regulations on Assisted Reproductive Technology’ prohibit medical institutions and medical personnel from engaging in surrogacy.	Article 51 of the Law on the Protection of Women’s Rights and Interests stipulates women’s right to give birth, while Article 17 of the Population and Family Planning Law states that citizens have the right to give birth.
Analogy	Article 3 and Article 7 of the Regulations on Human Organ Transplantation prohibit the buying and selling of human organs but allow for organ donation.	Article 1,007 of the Civil Code prohibits the buying and selling of organs, meaning the prohibition of commercial use of organs (including the uterus). While allowing for non-commercial organ donation, does this imply permitting the non-commercial use of organs (including the uterus)?

The legal contradictions have sparked varied debates on the legitimacy of surrogacy itself, with these discrepancies in legal theories emerging as a significant factor influencing the legalization of surrogacy. Regulating surrogacy through separate sectoral laws lacks specificity, and the legal outcomes are not entirely satisfactory. Article 1,009 of the Civil Code prohibits engagement in medical and research activities related to human genes, human embryos, and others, contravening laws, public order, and good customs. As a comprehensive legal framework, this provision is implemented through legal techniques, and its ultimate legal effects will be subject to the test of time.

### The judicial dilemma in determining surrogacy parent–child relationships

Court judgments undergo a dual examination, involving both legal and societal assessments. Judges, in their decision-making process, must adhere to the law while also staying attuned to public sentiments. Ultimately, any ruling has to withstand public scrutiny, encompassing moral judgment beyond mere legal evaluation.

Surrogacy disputes typically revolve around confirming the parent–child relationship of the surrogate child, specifically determining parental identity. Existing theories present four main perspectives (refer to Table 2).

- The Birth Theory: The birth mother as the legal mother, as seen in Australia (Constantinidis and Cook, 2012), Sweden (Vandenberghe, 2023), and the U.S. state of Washington (Hinson and McBrien, 2011).
- The Bloodline Theory: Parent–child relationships are determined based on the child’s bloodline, as observed in the UK (Crawshaw et al., 2012).
- The Agreement Theory: Parent–child relationships are determined based on surrogacy agreements, as observed in the US states of Arkansas, California, and Ohio (Hinson and McBrien, 2011).
- The Best Interests of Children principle: Legal parentage is determined based on what is deemed most conducive to the child’s well-being and development, as practiced in the US state of New Jersey (Ruth, 2015).
- The Adoption Theory (Liu, 2016b): Assumes the parent–child relationship of surrogate children based on legal adoption relationships, as observed in the US state of Nevada (Hinson and McBrien, 2011).

**Table 2:** Comparison of various theories on surrogacy parentage determination.

Theories	Birth	Bloodline	Agreement	Best interests of children
Advantages	Children have a recognized parent–child relationship from birth	Respecting the natural facts.	Aligns with the purpose of surrogacy and Respects freedom of contract	Comply with the convention on the rights of the child
Disadvantages	Acting against the purpose of surrogacy may lead to litigation with the intended parents	Unable to determine in the case of egg donation	If the intended parents violate the surrogacy agreement and abandon custody, the surrogate child becomes an orphan.	The intended parents may not have a biological relationship or parenting experience with the child and Legal proceedings may entail litigation costs.

Due to the absence of unified international surrogacy laws, decisions regarding surrogacy disputes vary from country to country and region to region, leading to divergent outcomes. For instance, in the UK, conflicting judgments and results have been documented (Yuan and Luo, 2016). In addition to the four mainstream theories outlined above, there is also the Adoption Theory. Its central concern revolves around the possibility that one or both intending parents may have a biological relationship with the surrogate child but are still required to ‘adopt’ their own child, a scenario perceived as illogical. Consequently, it garners minimal support, and this paper does not extensively explore it.

Each of the aforementioned theories has its merits, and none can be conclusively deemed as the most scientifically sound. The mainstream viewpoint suggests prioritizing biological relationships as the primary basis for determining parentage in surrogacy cases, supplemented by the principle of the child’s best interests. Specifically, the parents of the surrogate child are determined based on the genetic sources of the child. If both the biological father and mother are identifiable, but neither or both wish to assume custodial responsibilities, the determination of custody is made according to the best interests of the child. The current legal spouse of the custodian becomes the other legal parent of the child.

Given the significant costs associated with surrogacy, intended parents engaging in surrogacy arrangements are typically financially affluent and have a strong desire to care for the child. Moreover, the intending parents may share a genetic relationship with the child. From both subjective and objective perspectives, intending parents are more likely to secure custody of the child based on the child’s best interests. While this approach is relatively effective in resolving conflicts in determining the parent–child relationship in surrogate cases, its applicability is limited in addressing issues related to gestational surrogacy, where there is no genetic relationship between the surrogate child and the intended parents.

## Basic approaches to regulate surrogacy parental relationships

### Align with the legal principles of the civil code of China

#### Best interests of the child

Article 1073 Where an objection to maternity or paternity is justifiably raised, the father or mother may institute an action in the people’s court for affirmation or denial of the maternity or paternity.

Where an objection to maternity or paternity is justifiably raised, a child of full age may institute an action in the people’s court for determination of the maternity or paternity.

According to Article 1,073 of the Civil Code of China, the process of confirming the parent–child relationship in surrogacy unfolds as follows: Upon the child’s birth, the surrogate mother is acknowledged as the legal mother of the surrogate child, and her spouse is recognized as the father. If the surrogate mother’s spouse initiates a Denial Action in court, disavowing the parent–child relationship with the child, the husband of the commissioning party can claim to be the father of the surrogate child based on a genetic relationship, and his wife assumes the role of a “stepmother.” This becomes particularly pertinent in the context of gestational surrogacy, where, despite the pregnant and delivering surrogate mother lacking a genetic link to the child she brings into the world and potentially lacking the same emotional connection as the commissioning party’s wife, she is legally designated as the child’s mother. Simultaneously, her husband—a male with no biological ties to the child—automatically assumes the legal role of the child’s father. Navigating such a family dynamic is evidently suboptimal for the child.

The legal relationship between a stepmother and stepchildren is officially recognized as a mother-children relationship. However, a family consisting of a father and a “stepmother” may not provide the most conducive environment for a child’s upbringing. The child may always be aware that their biological mother is someone else. If the surrogate mother is unwilling to relinquish custody of the child to the intending couple who opted for surrogacy and instead prefers to co-parent with her husband, the child must navigate the intricate family dynamics of residing with the “mother and stepfather” on one side and confronting the “father and stepmother,” who played a pivotal role in their birth but remain an unfulfilled desire for cohabitation, on the other. Such complex family relationships are equally detrimental to the child’s overall development.

Therefore, it is only by establishing a clear legal definition of the parent–child relationship between surrogate children and either the surrogate couple or the intending couple that the law can truly adhere to the Best Interests of the Child principle outlined in the Marriage and Family Book of the Civil Code.

#### Balancing the relationships of surrogate parties

In establishing parent–child relationships, intending couples face a precarious situation due to the lack of direct legal regulations. Without assessing the potential benefits or drawbacks of the “complex” family dynamics resulting from surrogacy on a child’s development, the process of confirming surrogacy parent–child relationships puts intending couples in a passive position. They can only take on the roles of the child’s father and “stepmother” as a secondary option if both surrogate parents decline recognition as the child’s parents, initiate a denial of paternity lawsuit, and receive court confirmation.

In this scenario, even though intending couples incur substantial expenses for surrogacy to welcome surrogate children, they still face significant risks of not securing parental rights over the child. If these risks and the underlying interests are not clearly and explicitly regulated, they may transform into a source of criminal activities. Particularly in cases of partial surrogacy, where the surrogate mother and the surrogate child share a genetic connection, the surrogate mother might naturally assume the role of the child’s mother, potentially becoming a “breach party” without considering the intending couple, who may have already invested considerable resources. In such situations, it is crucial for the law to regulate and balance the relationships between the intending couple and the surrogate child by precisely defining parent–child relationships, thereby safeguarding the vulnerable surrogate parties (Zhang, 2019).

#### Protecting the vulnerable within marriage and family

The legislative intent behind family law primarily centers on safeguarding the interests of those who are vulnerable within familial relationships and fostering gender parity. It is irrefutable that the ambit of assisted reproductive technologies (ART) extends to encompass *In Vitro* Fertilization (IVF) and Surrogacy, two modalities with distinct orientations; the former predominantly caters to male infertility, whilst the latter to female infertility. ARTs provide a means through which childbirth can be facilitated, offering a mechanism for the legal acknowledgment of challenges pertaining to male infertility. Conversely, in the context of female infertility, whilst such technologies similarly enable childbirth, there exists a notable deficit in legal safeguards. An exclusive focus on the male partner’s interests, to the detriment of the female partner’s, would result in a skewed protection mechanism, undermining the foundational principles of family law aimed at bolstering female protection. An illustrative instance of this gender-protective stance is encapsulated in the “domestic labor compensation” scheme, as delineated in Article 1,088 of the Chinese Civil Code. This provision exemplifies a legislative effort imbued with a gender protection bias, bearing considerable practical import.

Article 1088 Where one of the spouses performs more duties in bringing up children, taking care of the elderly or assisting the other spouse in his or her work, that spouse shall have the right to claim compensation from the other spouse in the case of divorce, and the other spouse shall make compensation. The specific arrangements shall be agreed upon by both parties. If they fail to reach an agreement, the people’s court shall make a judgment.

The import of embedding Marriage and Family Law within the Civil Code is manifest in its enhanced safeguarding of the rights and interests of those vulnerable within familial contexts (Yang, 2020). Within the dynamics of matrimonial bonds, it is frequently observed that women are placed in a less advantageous position relative to their male counterparts. The inability to conceive places women at a heightened risk of facing divorce compared to men. Consequently, it is imperative that legal protections, inherently biased toward the female gender, are instituted for women engaging in surrogacy. The Civil Code ought to recognize the legal status of children born through surrogacy, elucidating the nature of their parental relationships with clarity.

#### Establishing a more reasonable parent–child relationship

Upon scrutinizing the prevailing methodology for ascertaining parent–child affiliations in China, which is steered by the doctrine that “the birth mother is deemed the legal mother,” it becomes evident that the provenance of gametes plays a secondary role in influencing these bonds. The crux of the matter hinges upon the gestational carrier – the individual who undergoes the pregnancy. In essence, the law recognizes the woman who undergoes pregnancy and subsequently gives birth as the legal mother of the child, and her husband is recognized as the father, except in instances where paternity is disputed. Nonetheless, this principle reveals itself to be markedly insufficient and impractical when applied to the context of surrogacy.

The surrogate mother’s principal impetus for embarking on pregnancy and childbirth frequently transcends the mere aspiration for a child, encompassing motives such as financial remuneration. The inception of the surrogate child does not stem from a conscious choice or anticipation by the surrogate mother, nor does she entertain an active intent to embrace the mantle of “motherhood.” In contrast, her partner is attributed the title of “father” purely by virtue of the matrimonial bond with the surrogate mother, despite his recognition of the child’s lack of biological connection to him. The constitution of the parent–child bond under these premises is devoid of logical foundation. Therefore, the enactment of precise legal statutes is imperative to imbue the parent–child dynamic within surrogacy with rationality.

### Building on the existing two methods of “quasi-blood relationship” determination

In accordance with the Civil Code of China, there are primarily two legal drafting approaches for individuals serving as parents and children despite lacking a biological relationship.

#### Step-parent and step-child relationship

In instances where a child’s parent has passed away and the surviving parent enters into a subsequent marriage, thus establishing a new marital familial bond, the creation of an actual caregiving dynamic between the new spouse and the child is legally acknowledged as a “step-parent and step-child relationship” within China. Pursuant to Articles 849 and 882 of the Civil Code, the determinative factors for identifying a step-parent and step-child bond hinge on the formation of a caregiving connection, necessitating both a subjective intention and the execution of caregiving duties.

Initially, the new spouse is required to voluntarily embrace their partner’s child from before the marriage and actively participate in a parent–child dynamic. Subsequently, the parents are obligated to undertake responsibilities including caregiving, nurturing, and educating the child. Upon the establishment of the step-parent and step-child bond, the demise of the biologically connected parent does not affect the continuation of the parent–child relationship between the non-biological parent and the child.

#### Adoption relationship

In line with Article 884 of the Civil Code, adoption is legally formalized once the prospective adoptive parents fulfil the stipulated adoption criteria and proceed with registration at the civil affairs department of the people’s government at the county level or higher. A legitimate adoption process constitutes a prerequisite for adoption, implying that an adoption lacking registration and the formalization of an adoptive relationship is deemed invalid and lacks legal safeguarding. Within China, the prerequisites and procedures for adoptive parents are rigorously defined, underscoring the imperative to strike a balance between the ease of adoption and the prospective living conditions for the adoptees. The imposition of stringent adoption requisites is paramount to safeguarding the welfare of minors (Zhou, 2022).

### The two types of surrogacy parent–child relationships should be regulated separately

The prevailing Marriage Law, as enshrined within the Civil Code, delineates the aforementioned two types of non-genetic familial bonds, termed Quasi-blood Relations. Yet, it fails to address the intricacies of parent–child relationships concerning children born through surrogacy. Surrogacy involves embryos originating from different egg cell sources, culminating in two distinct forms as outlined in Table 3: gestational surrogacy and traditional surrogacy. The biological and genetic connections significantly influence the relationship between the surrogate mother and both the surrogate fetus and the child post-birth. Consequently, the formulation of parent–child relationships for these two divergent surrogacy modalities necessitates individualized consideration.

**Table 3:** Comparison between *in vitro* fertilization and surrogacy.

Type		Sperm source	Egg source	Pregnancy subject	Legal or not
*In vitro* fertilization		Sperm donors	Mother	Mother	Yes
		Father	Mother	Mother	Yes
Surrogacy	Gestational surrogacy	Father	Surrogate mother	Surrogate mother	No
	Traditional surrogacy	Father	Mother	Surrogate mother	No

#### Full surrogacy

In full surrogacy, the fertilized egg is introduced into the surrogate mother’s body utilizing medical technologies to facilitate pregnancy and childbirth. With regards to full surrogacy, wherein the child’s genetic makeup derives exclusively from the prospective parents, the issue arises as to whether the intending couple can affirm their biological connection with the child via conclusive paternity test outcomes, instigate legal actions, and thereby directly ascertain the parent–child and custody dynamics through judicial means. Alternatively, they might be required to undertake the adoption procedure to formalize a legal parent–child relationship.

Both approaches incur considerable financial expenditures and the latent risk of the surrogate mother contravening the agreement and declining to relinquish the child. Even if the prospective parents successfully traverse the protracted legal journey to validate the parent–child relationship—a process that may span months or years—the child will have already forged an initial bond and cemented a stable, pre-existing familial relationship within the surrogate mother’s household. At this juncture, altering the child’s initial familial setting is bound to adversely affect their emotional health and holistic development.

In an alternative scenario of full surrogacy, the procedure employs eggs from an anonymous donor to create the embryo of the surrogate child. This embryo is then fertilized with sperm from the prospective father, following which the surrogate mother gestates and delivers the child. This circumstance presents a pragmatic paradox: albeit the surrogate child and the surrogate mother share no biological ties, the genetic lineage from the maternal side remains undisclosed. The surrogate mother, serving as the birth mother, harbors no aspirations to rear the child. In contrast, the intending mother, who is keen on parenting the child, encounters legal hurdles that obstruct her path to becoming the child’s legal mother.

The interaction between the prospective couple, emotionally invested and longing for the child, and the surrogate household, disengaged during the pregnancy and disinterested in parenting the child, unmistakably favors the former for the welfare of the child. Nonetheless, prevailing legal statutes stipulate that the child ought to reside with the surrogate family, thereby conflicting with the paramount principle of acting in the child’s best interests.

#### Partial surrogacy

In the scenario of partial surrogacy, commonly referred to as traditional surrogacy, wherein the surrogate mother contributes her own egg for the conception of the child, the spouse of the intending father, who jointly consents to surrogacy to enable childbirth, is legally acknowledged solely as the child’s stepmother. This presents a paradox, as she is the primary influencer in the decision-making process regarding the child’s birth, invests considerable emotional commitment, and actively undertakes the duties of nurturing and educating the child, thereby rightfully assuming the maternal role.

In accordance with the provisions outlined in the Civil Code concerning the custody of children following parental separation, it can be inferred that in cases where the biological parents are not cohabiting, the child should primarily reside with the surrogate mother’s family during the breastfeeding phase. As the child progresses through developmental stages, custody may transition to the father’s family. However, within the context of surrogacy, the surrogate mother neither expects nor desires to raise the child subjectively, and objectively, her living conditions may not inherently surpass those of the intending couple who initiated the child’s conception. Consequently, for the surrogate child, residing with the intending couple’s family post-birth better aligns with the principle of serving the child’s best interests.

On the contrary, the designation of stepmother given to the intending wife could potentially evoke resentment in the child, thereby detrimentally affecting the child’s development.

## Legislative proposals for regulating surrogacy parent–child relationships in China’s civil code

Numerous international precedents exist that directly recognize parent–child relationships in surrogacy cases. One of the most notable instances can be found in Article 5 of the Uniform Parentage Act in the United States. According to this provision, if a wife undergoes artificial insemination using sperm from a third party, and her husband consents under the supervision of a licensed physician, the husband is legally acknowledged as the biological father of the child. Similarly, if the husband’s sperm is used for *in vitro* fertilization with eggs from a donor and subsequently implanted in the wife, the resulting child is considered to be born within the bounds of marriage. This legal provision explicitly recognizes parent–child relationships in cases involving sperm donation and *in vitro* fertilization.

However, in scenarios resembling full surrogacy, where fertilized eggs are implanted in a woman other than the wife, the biological father cannot be directly acknowledged as the father, and the wife cannot be directly acknowledged as the mother under the existing legal framework.

Surrogacy children are acknowledged to hold the status of offspring born within wedlock, a perspective increasingly recognized by scholars (Xiao, 2019). To validate the parent–child relationship in surrogacy cases, it is imperative to initially nullify the parental bond between the surrogate child and the surrogate mother and her spouse. Subsequently, the establishment of the parent–child relationship between the surrogate child and the intending couple must be confirmed. The dissolution of the parent–child relationship between the surrogate child and the surrogate mother’s spouse can be addressed through the Paternity Denial Lawsuit system outlined in the Civil Code. However, the legal clarification regarding the establishment of the parent–child relationship between the surrogate child and the intending couple necessitates explicit legal provisions.

### Denying the parent–child relationship between the surrogate mother and her spouse and the surrogate child

When it comes to surrogate children, establishing the mother–child relationship solely based on the birthing theory lacks rationality. While determining parent–child relationships based on childbirth might seem straightforward, it may not encompass all relevant factors. In most countries globally, parent–child relationships are determined through a presumption method, which, being presumptive, may not always reflect the true circumstances. This is why Article 850 of the Civil Code introduces the Paternity Denial Lawsuit system, enabling interested parties to challenge the parent–child relationship. The primary basis for contesting the father-child biological relationship is the absence of a genetic link. Similarly, if a woman merely carries a child for someone else, the mother–child relationship can be subject to challenge.

#### Full surrogacy

The Marriage and Family Book of China’s Civil Code is regarded as the legal framework that best embodies gender equality.

The second paragraph of Article 1041: A marriage system based on freedom of marriage, monogamy and equality between man and woman is applied.

Men and women are entitled to equal rights regardless of physiological disparities. Hence, in the realm of full surrogacy, where the surrogate does not contribute the egg and lacks any genetic link with the surrogate child, she can, akin to a male, assert the denial of her maternal status based on scientific evidence demonstrating the absence of a genetic relationship. Conversely, the wife of the commissioning party, who shares a genetic bond with the surrogate child, assumes the role of the mother.

In the context of full surrogacy, there is no necessity for the surrogate mother to pursue a paternity denial lawsuit for the intending party’s wife to establish a parent–child relationship with the surrogate child. Given the absence of a genetic link between the surrogate mother and the child, and considering that pregnancy and childbirth are integral components of fulfilling the surrogacy agreement, the legal recognition of the mother–child relationship between the intending party’s wife and the surrogate child can be directly governed by the law. The initiation of a paternity denial lawsuit by the surrogate mother does not impact the mother–child relationship between the intending party’s wife and the surrogate child. Should the surrogate mother refuse to deliver the child and assert a mother–child relationship based solely on childbirth, she would bear the consequences of breaching the contract. Moreover, she risks losing a future dispute over parent–child relationships, particularly if the intending party’s wife provides positive results from a paternity test.

#### Partial surrogacy

In cases of partial surrogacy where the surrogate mother shares a genetic bond with the surrogate child, the grounds for a paternity denial lawsuit can solely rely on a valid surrogacy agreement. This lawsuit petitions the court to nullify the mother–child relationship between the surrogate mother and the child, and once terminated, the surrogate mother is barred from seeking court affirmation of their relationship anew. Upon the court’s confirmation of the termination of the mother–child relationship between the surrogate mother and the child, the intending party’s wife can legally assume the role of the child’s mother. To uphold the interests of both parties involved in surrogacy and foster stability in the familial bond of the surrogate child from an early stage, it is recommended to impose a time constraint on the initiation of paternity denial lawsuits by surrogate mothers. For instance, this could involve limiting the timeframe for the surrogate mother and her spouse to file a paternity denial lawsuit to within 30 days following the child’s birth.

The initiation of a paternity denial lawsuit by the partial surrogate mother is a necessary step in establishing the parent–child relationship in surrogacy between the intending couple and the surrogate child. This necessity arises because the surrogate mother and the surrogate child have a genetic link, and the occurrence of pregnancy and childbirth automatically designates her as the child’s mother. However, as pregnancy and childbirth are integral to fulfilling the surrogacy agreement, the surrogate mother retains the discretion to decide whether to commence a paternity denial lawsuit, thereby determining whether to confirm a mother–child relationship with the surrogate child. Should the partial surrogate mother opt not to seek the court’s intervention to annul the mother–child relationship with the surrogate child, she retains her status as the child’s mother. Nevertheless, this does not impact the surrogate child’s standing as the lawful offspring of the intending couple.

Under usual circumstances, when the surrogate mother instigates a paternity denial lawsuit against the surrogate child based on the surrogacy arrangement, the court is inclined to uphold the surrogate mother’s spouse in severing the familial tie with the surrogate child. However, exceptions exist. If, prior to the child’s birth, the intending couple becomes incapable of caring for the surrogate child due to circumstances such as their demise or the forfeiture of their legal capacity, and such care by them is deemed detrimental to the child’s welfare, conflicting with the principle of the “best interests of the child, “the court must evaluate the situation. In necessary instances, the court may curtail the surrogate mother’s entitlement to initiate a paternity denial lawsuit. Naturally, an adverse judgment outcome may only ensue if the commissioning couple loses their caregiving capacity; otherwise, the court should endorse the surrogate mother’s spouse in terminating the parent–child relationship with the surrogate child.

### Confirming the parent–child relationship of the intending couple

Should the surrogate mother be willing to relinquish custody of the surrogate child to the intending couple, the pertinent legislative concern is whether the parent–child relationship between the intending couple and the surrogate child can be legally recognized under the Civil Code.

#### Full surrogacy

Given that in full surrogacy scenarios, the surrogate mother and the surrogate child lack any genetic connection, it is proposed that the law expressly declare the wife of the intending party as the mother of the child born through full surrogacy. This provision offers the benefit of simplifying the process for recognizing the parent–child relationship in full surrogacy cases. Establishing the parent–child relationship between the intending couple and the surrogate child would only necessitate positive results from a paternity test, obviating the need for an adoption procedure. This streamlined approach reduces the intricacy and associated expenses of the recognition process, facilitating a swifter transfer of custody to the intending couple. Essentially, once the surrogate mother renounces the mother–child relationship with the child, the intending couple can petition the civil affairs department to become the child’s legal guardians based on a valid surrogacy agreement and positive paternity test results, without requiring additional confirmation procedures.

### Partial surrogacy

In cases of partial surrogacy, where the surrogate mother provides the egg, she is deemed the biological mother of the surrogate child. If the surrogate mother instigates a paternity denial lawsuit and secures court affirmation, the mother–child relationship with the child is terminated, thereby enabling the intending couple to establish a parent–child relationship with the child. However, if the surrogate mother refrains from filing a paternity denial lawsuit, the husband of the intending party is acknowledged as the biological father of the child. The wife of the intending party, predicated on her marital bond with the child’s father, assumes the role of stepmother, while the child is recognized as the marital offspring of the intending couple.

It is imperative for the law to stipulate that children born through surrogacy should reside with their father and his spouse immediately after birth, rather than mandating that they live with the birth mother until a certain stage before transferring custody to the father. This approach is more beneficial for the child’s holistic well-being and development.

## Conclusion

This paper presents legislative proposals for the judicial interpretations of the Chinese Civil Code, aiming for Chinese law to comprehensively regulate the issue of surrogacy, particularly concerning parent–child relationships arising from surrogacy.

Children born through surrogacy officially recognize the intending party’s husband as the father, with the child being legally regarded as the marital offspring of the intending couple. In the scenario of full surrogacy, the intending party’s wife is formally acknowledged as the mother of the child born through surrogacy. Regarding partial surrogacy, the establishment of the mother–child relationship occurs between the child and the intending party’s wife when the surrogate mother willingly relinquishes her parental connection with the child. Consequently, children born through surrogacy, similar to those born through other assisted reproductive technologies, should receive legal recognition and be granted the legal status of marital children.

The regulation of surrogate parent–child relationships can be clearly outlined, but the decision to legalize surrogacy requires careful consideration. This involves a comprehensive assessment of factors such as economic capability, international reputation, and population demographics. Additionally, a critical examination of supporting structures and the adaptability of the social environment to potential adverse effects is crucial. While the prevailing tendency leans toward the legalization of surrogacy, it should unfold gradually, without haste.

## Data availability statement

The original contributions presented in the study are included in the article/supplementary material, further inquiries can be directed to the corresponding author/s.

## Ethics statement

Ethical approval and written informed consent were not required for this study in accordance with the local legislation and institutional requirements.

## Author contributions

WY: Writing – original draft, Writing – review & editing. JF: Writing – original draft, Writing – review & editing.

## Footnotes

^1^ https://www.who.int/zh/news/item/04-04-2023-1-in-6-people-globally-affected-by-infertility

^2^ https://wenshu.court.gov.cn/website/wenshu/181217BMTKHNT2W0/index.html?pageId=cc6d2c72f3d14b523525cf136adb856b&s21=%E4%BB%A3%E5%AD%95
